# Integrative use of DNA barcode and morphology reveal high level of diversity in the ornamental fish on the lower Amazon basin

**DOI:** 10.1371/journal.pone.0316455

**Published:** 2024-12-30

**Authors:** Elciane Araújo de Freitas, Dayana Batista dos Santos, Charles Samuel Moraes Ferreira, Cárlison Silva-Oliveira, Grazielle Fernanda Evangelista-Gomes, Ivana Barbosa Veneza

**Affiliations:** 1 Undergraduate Course in Aquaculture Engineering, Federal University of Western Pará, Monte Alegre, Pará, Brazil; 2 Laboratory of Applied Genetics, Coastal Studies Institute, Federal University of Pará, Bragança, Pará, Brazil; 3 Federal University of Western Pará, Postgraduate Program in Society, Nature and Development, Santarém, Pará, Brazil; Pontificia Universidade Catolica do Rio Grande do Sul, BRAZIL

## Abstract

The Amazon basin is the world’s largest hydrographic basin, in terms of both its total area and its species diversity, with more than 2,700 species of fish. Despite this diversity, the data available on the fish fauna of the Amazon basin are still relatively scant and incomplete, in particular from the streams and floodplain lakes of the lower Amazon, which may contain a large proportion of the still undescribed species of the basin. Many of these species are expected to be of interest to the ornamental fish market. The investigation of the diversity of potential ornamental fish using molecular tools is even more limited. Given this scenario, the present study employed DNA barcoding to investigate the diversity of ornamental fish found in two streams and a floodplain lake of the lower Amazon. The mitochondrially encoded cytochrome c oxidase I (MT-CO1) molecular marker was used to identify the taxa, in combination with morphological keys. A total of 51 ornamental species were identified, representing 13 families and three orders. A majority of the species were found at only one of the sampling points, which indicates that the distribution of the species is influenced by ecological factors. The most speciose order was the Characiformes, followed by the Cichliformes and Siluriformes, while the family with the greatest diversity of species was the Acestrorhamphidae (31.3% of the total number of species), followed by the Cichlidae (27.4%), and the Lebiasinidae (9.8%). One specie was registered in the region of the lower Amazon for the first time, and evidence was found of the possible existence of species not formally described of *Aphyocharax*, *Astyanax*, *Apareiodon* and *Hemigrammus*.

## Introduction

The Amazon basin is the world’s largest hydrographic basin [1], with a total area of 7,050,000 km^2^, and is also the region with the greatest diversity of aquatic organisms, in particular freshwater fish, of which, more than 2,700 valid species have been documented to date, with many others yet to be described [2, 3]. This fauna represents approximately 15% of all freshwater fish species described up to now [4].

Many of these species are highly valued on the ornamental fish market [5], which has grown exponentially in recent years, and currently represents a global industry that generates sales of US$15–30 billion annually [6]. Estimates indicate that the global ornamental fish trade will grow at a mean rate of 8.5% per year, between 2022 and 2030 [7].

This growing ornamental fish trade is in constant search for more fish, to meet demand, and a greater variety of species, to attract buyers. By 2016, international exports of ornamental fish had grown by 84.8% in comparison with 2001 [5, 8] (International Trade Centre, 2018; Rezende; Fujimoto, 2021). During this same period, Brazilian exports increased by 103.7%, which raised Brazil to the level of a major ornamental fish exporting country [5, 8].

The vast majority of these exports (98%) originate from the Amazon region, in particular, the Brazilian states of Pará and Amazonas, which are the country’s principal exporters [5, 8]. Between 2013 and 2017, Pará was responsible for 73.4% of the Brazilian exports of ornamental fish [5].

Despite the known diversity of the fish species found in the Amazon basin, which is determined, in part, by the extreme complexity of this drainage system [9], it is assumed that the true diversity of the basin’s fish is still grossly underestimated. This assumption is based on both the large number of species that are being described annually [10], and the relatively limited scientific sampling available for most of the vast area of the basin [11, 12].

This enormous diversity of fish is not restricted to the basin’s major rivers, such as the Amazon, Negro, and Madeira, but extends throughout its vast network of smaller streams, known locally as *igarapés*, watercourses of the first to third order, which consist of shallow, long channels that penetrate deep into the forest [13]. It seems likely that much of the still undescribed species diversity is found in these relatively cryptic and poorly-known environments [14].

The lakes of the Amazon floodplain, which are an important component of this complex drainage system, are highly dynamic environments that are quite distinct from its river and streams [15]. Even so, these habitats also tend to have a diverse fish fauna, albeit relatively poorly-known and neglected by investigators [16–18].

The total diversity of the freshwater species found in the Amazon basin is still unclear, and there are many inaccurate reports of the number of species present in the rivers of this basin, including the region of the lower Amazon [19]. Given these shortcomings, molecular tools, such as the DNA barcode, provide powerful instruments for the investigation of the taxonomy and diversity of a number of different animal groups, and can enable the correct identification of fish species, especially when these species are poorly-known or morphologically similar [20].

In this context, the present study surveyed the ornamental fish fauna of two streams and a lake located in the region of the lower Amazon River, using DNA barcodes in combination with morphological criteria.

## Material and methods

### Ethics declaration and authorization for the collection and transportation of specimens

The collection and transportation of the fish specimens were registered in the Brazilian Biodiversity Authorization and Information System (SISBIO), under license number 86127–1. Once captured, fishes were anesthetized and then euthanized by immersion in a lethal eugenol solution at a concentration of 180 mg.L-. The procedures adopted for the euthanasia of fish were approved by the Ethics Committee for the Use of Animals of the Federal University of Western Pará (CEUA-Ufopa), through protocol number 1020220223.

### Characteristics of the study area

**Fig 1:**
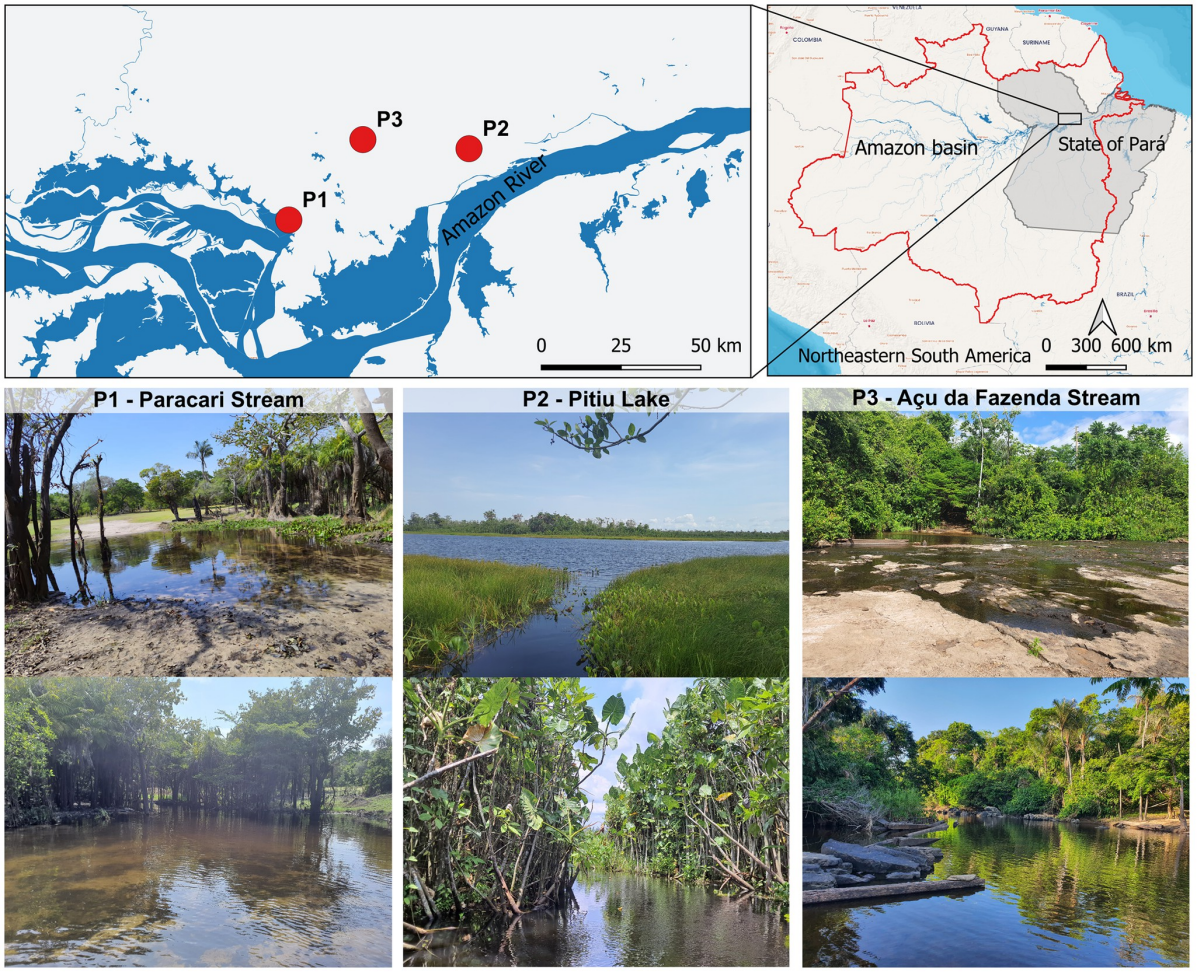
Sampling locations. Spatial arrangement of the three sampling points surveyed on the floodplain of the lower Amazon (upper) and the typical landscape of each site.

The present study focused on tree geomorphological habitat types in the lower Amazon River: floodplain lake, terra firme stream, and shield stream (Fig 1). Paracari Stream (point 1 –P1 in Fig 1) has a moderate flow of water, with an abundance of trunks, branches, and leaves resting on its sandy bottom and around its margins. The middle and upper portions of the stream are covered by the forest canopy. The shade and continuous flow of water in these environments reduce their primary productivity, which is apparent from the transparent water. There are some beds of floating macrophytes, primarily *Salvinia minima* and *Eichhornia crassipes*.

Pitiú Lake (P2 in Fig 1) is a lacustrine environment, with calm and dark water surrounded by dense vegetation around its margins, and large quantities of branches, dead leaves, and tree trunks. Some lake areas are covered by forest canopy, while others are completely exposed to sunlight. In other parts, the accumulation of organic matter has depleted the oxygen levels of the water, which leaves some fish gasping for air at the surface. The availability of oxygen varies considerably between the more open areas and those with dense vegetation. The bottom of the lake is muddy, and beds of both floating and submerged macrophytes have formed throughout its area, in particular floating reeds, water hyacinth (*Eichhornia crassipes*), fanwort (*Cabomba caroliniana*), water lettuce (*Pistia stratiotes*), and salvinia (*Salvinia minima*). This lake is an extremely complex environment, which encompasses an enormous heterogeneity of habitats.

Açu da Fazenda Stream (P3 in Fig 1) is a typical shield tributary dominated by rapids, with a sandy substrate and many large rocks, which are arranged in linear formations in some places. The water is transparent and, while the stream is surrounded by vegetation, it does not cover the body of water, permitting a greater penetration of sunlight.

### Sampling, classification and cataloging of the specimens

**Fig 2:**
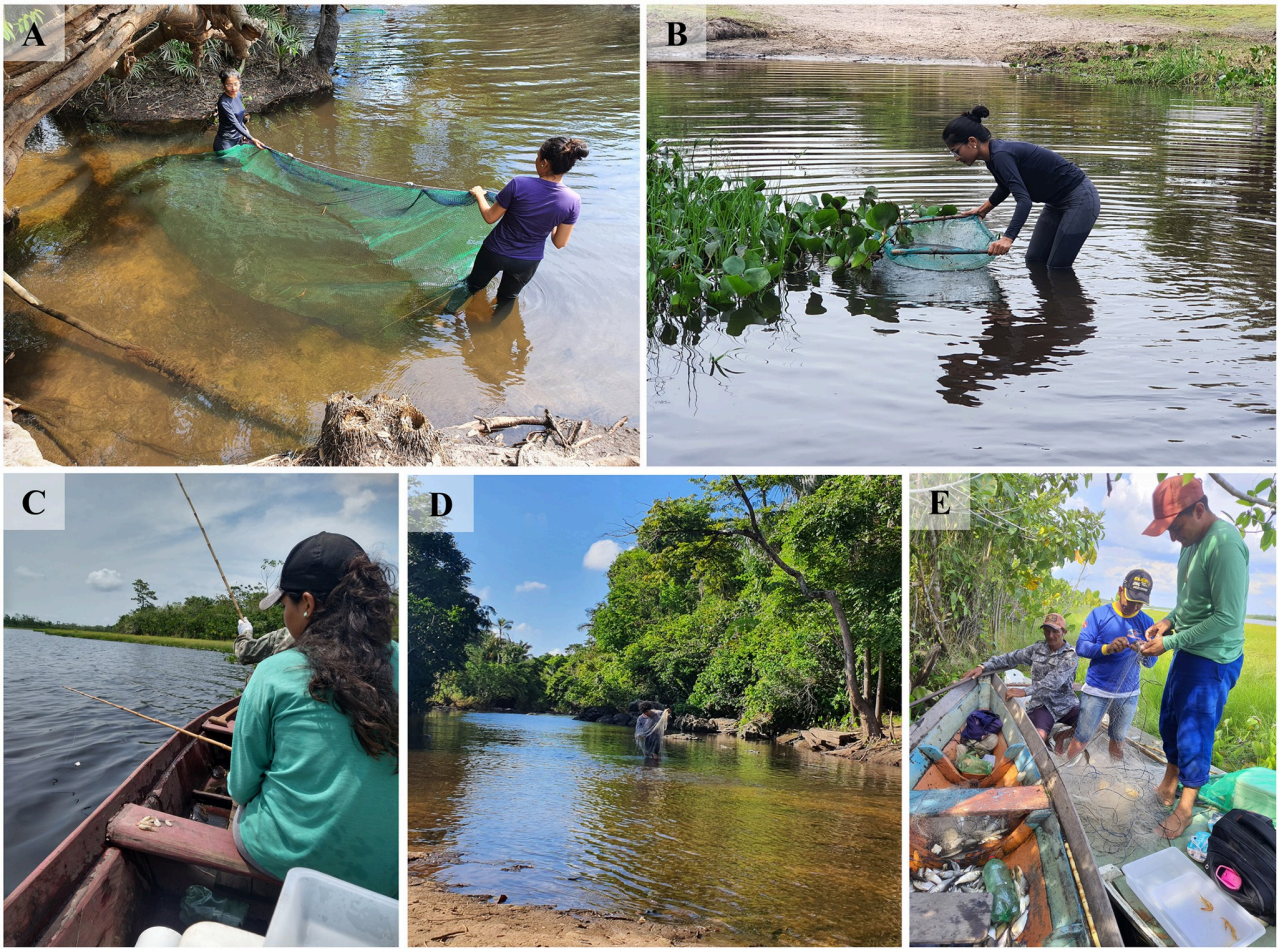
Types of gear used to collect fish specimens at the three points surveyed in the present study. **A.** Dragnet; **B.** Sieve; **C.** Rod and line; **D.** Cast net; **E.** Gillnet.

Two samples were collected at each point, in October and November 2022, primarily during the morning. At the two streams (Paracari and Açu da Fazenda), the fish were collected using a manual trawl or dragnet, with a 5 mm mesh, 4.5 m long and 1.5 m high, for approximately 50 m (Fig 2A), a sieve, also with a 5 mm mesh (Fig 2B), rod and line (Fig 2C), and a cast net, with a 20 mm mesh (Fig 2D). The dragnet was deployed within a stretch of approximately 50 m that was delimited within the stream, a procedure that has been employed in a number of previous surveys of the fish fauna of the Amazon region [21, 22]. At Pitiú Lake, the dragnet was replaced by a gillnet with a 15 mm mesh (Fig 2E), given the much greater depths found in this lake, in comparison to the streams.

Once captured, fishes were anesthetized and then euthanized by immersion in a lethal solution of Eugenol (180 mg/L). The species were classified as ornamental fish when they presented features considered to be attractive, such as bright and varied coloration, and small size–no more than 15 cm–as defined by [15], as well as elegant body and fin shapes or an overall unusual appearance [5, 23].

Once classified in terms of their potential as ornamental fish, the specimens were assigned catalog numbers, photographed, and then stored on ice in a styrofoam cooler box for transportation to the laboratory on the Monte Alegre campus of the Federal University of Western Pará (UFOPA). In the laboratory, biological tissue samples were extracted from the caudal fin, basihyal or dorsal region of the specimens. These samples were stored in cryogenic microtubes containing 70% ethanol and frozen at a temperature of approximately -20ºC until being used for the molecular procedures described below.

Voucher specimens were preserved for each species, on average five individuals per species (when possible), with these specimens being fixed in 10% formalin and then conserved in 70% ethanol, before being deposited in the ichthyological collection of the UFOPA campus at Monte Alegre. Some of the fish included in the study from which we removed tissue were very small specimens (1–4 cm total length), and it was not possible to remove the biological tissue in a way that would adequately preserve its morphology for deposit in a collection. Therefore, some of the individuals from which we used tissue were not deposited, but rather another representative of the same species, which was properly morphologically identified. The codes of all individuals used in the study, both those with a witness specimen deposited in the collection and those that are only deposited in the biological tissue bank, are listed in S1 Table, in the column designated as “Catalog number”.

### Isolation, amplification, and sequencing of the genetic material

The molecular procedures were conducted in the Laboratory of Applied Genetics, on the Bragança campus of the Federal University of Pará. The total DNA was extracted using the NaCl protocol [24]. The extracted material was stained with GelRed^TM^ (BIOTUM)+bluejuice and then eletrophoresed in 1% agarose gel. Following electrophoresis, the gel was visualized under ultraviolet light to detect the isolate.

The primers proposed by [25]–FishF1 (5’-TCAACCAACCACAAAGACATTGGCAC-3’); FishR1 (5’-TAGACTTCTGGGTGGCCAAAGAATCA-3’); FishF2 (5’-TCGACTAATCATAAAGATATCGGCAC-3’); and FishR2 (5’-ACTTCAGGGTGACCGAAGAATCAGAA-3’)–were used to amplify the barcode portion of the MT-CO1 gene (mitochondrially encoded cytochrome c oxidase I).

The samples were amplified by Polymerase Chain Reaction (PCR), with a final volume of 15 μL, made up of: 2.4 μL of DNTP (1.25 mM), 1.5 μL of 10x buffer, 0.6 μL of MgCl_2_ (25 mM), 0.6 μL of each primer (10 pmol), 0.2 μl of Taq polymerase (5U.μL^-^), 0.6 μl of the total DNA, and purified water to complete the final reaction volume.

The amplification protocol consisted of: initial denaturation at 94°C for 5 minutes, followed by 38 cycles of denaturation at 94°C for 40 seconds, hybridization for 30 seconds, and extension at 72°C for one minute, with a final extension at 72°C for 3 minutes. The hybridization temperature was either 50°C, 54°C or 56°C, depending on the species.

The amplicons were purified with polyethylene glycol (PEG 8000), using the protocol described by [26], and then sequenced by the dideoxyterminal method of [27], using reagents of the Big Dye kit (ABI Prism TM Dye Terminator Cycle Sequencing Reading Reaction*–Thermo Fisher Scientific*, *USA*). The precipitated products were electrophoresed in an ABI 3500 XL capillary automatic sequencer (*Thermo Fisher Scientific*).

### Computer analyses

The sequences obtained were edited in BioEdit v.7.1.3.0 [28], and then aligned in CLUSTAL W, in the automatic mode [29], which is included in the editing program. A sequence library was established for each sampling point.

The specimens were identified based on the similarity of their MT-CO1 gene sequences with those obtained from the GenBank (NCBI—National Center for Biotechnology Information—http://www.ncbi.nlm.nih.gov) and BOLD (Barcode of Life Data Systems—http://www.boldsystems.org/) reference libraries, for comparison. Similarity was defined as a complete agreement of more than 98% between the nucleotides of a pair of sequences [30], this being the threshold adopted by both platforms used, to consider individuals included in the same species.

The sequences were also used to compile a phylogenetic tree to confirm the identification of the specimens, based on the topology of the tree, with the most closely-related clades corresponding to the species. Sequences of the target species available in the GenBank or BOLD databases were added to the database for the construction of the Neighbor-Joining (NJ) tree, using the Kimura 2-parameter evolutionary model [31], which was run in MEGA X [32], with the significance of the clusters being estimated using a bootstrap analysis with 1,000 pseudoreplicates [33].

For possible cryptic species, we calculated in MEGA X the genetic distance between and within the groups, using the distance p and 1000 bootstrap pseudoreplicates.

### Morphological identification

To complement the molecular identification, the specimens were also identified based on their morphological features, using either taxonomic identification keys [34–37] or consultations with specialists. Taxonomic classification of Characiformes follows [38].

Some specimens received provisional identification, which may even indicate insufficient research for some taxonomic groups or may be indicative of new species after more refined analysis. Thus, the abbreviation “sp.” (species) was used to name a species of a genus which could not be identified. When there is uncertainty about the identification (insufficient or damaged material), we use the abbreviation “cf.” which means “compare with”. When a species cannot be identified, but resembles another already described, it is considered that it must be a new species and the abbreviation “aff.” (from the Latin “affinis”) was used.

## Results

Overall, 145 sequences of the barcode fragment of the MT-CO1 gene were obtained from the specimens collected during the present study on the lower Amazon (BOLD access codes: AMZON001 –AMAZON145). These sequences had a mean length of 602 bps, which corresponded to those of 51 known species from 13 families and three orders.

**Fig 3:**
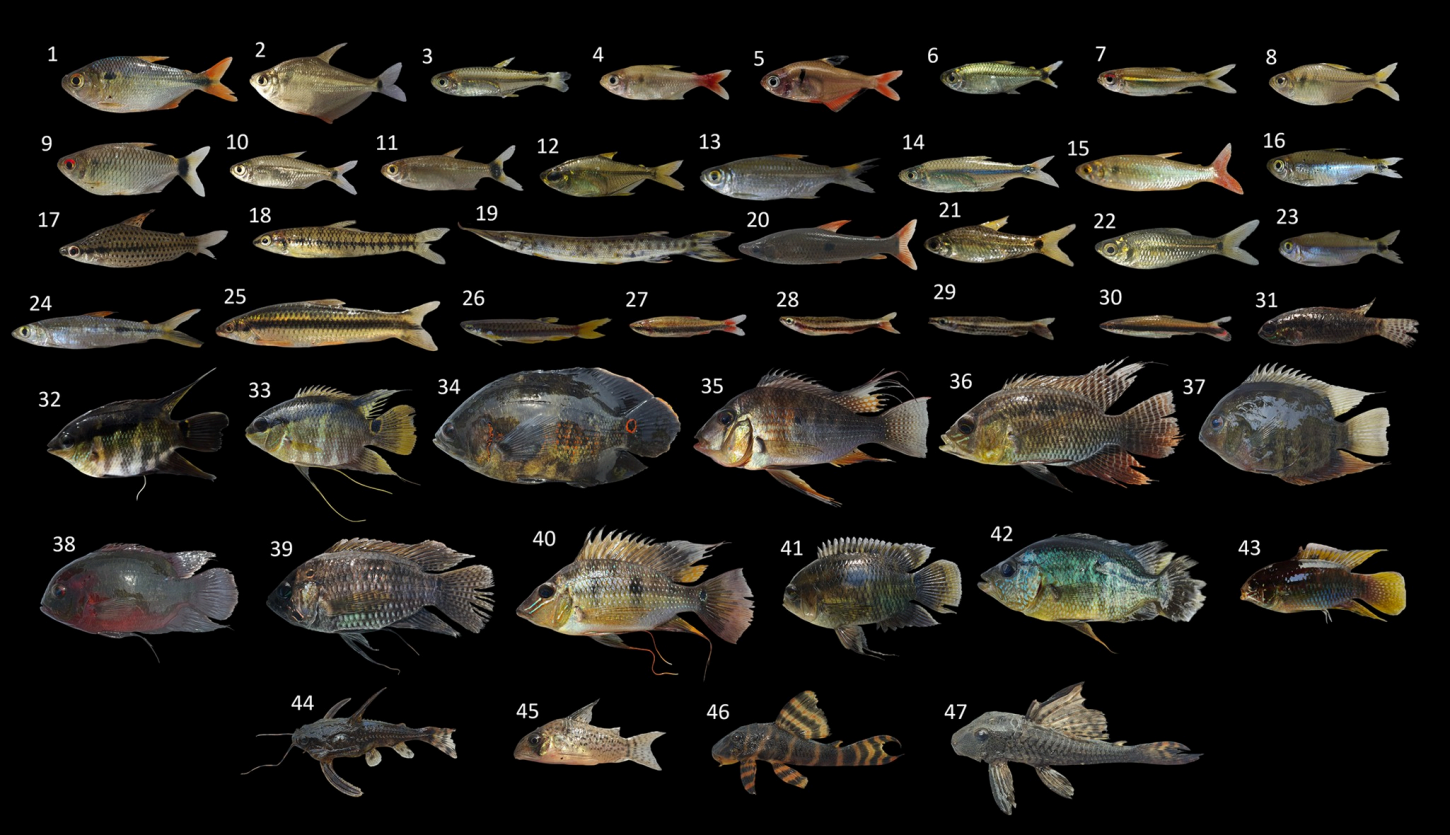
Some ornamental fish species collected from the three sampling points on the lower Amazon. 1—*Astyanax* aff. *argyrimarginatus*; 2—*Ctenobrycon spilurus*; 3—*Hemigrammus durbinae*; 4—*Hemigrammus stictus*; 5—*Megalamphodus* cf. *bentosi*; 6—*Hemigrammus* sp.; 7—*Hemigrammus analis*; 8—*Makunaima guianensis*; 9—*Bario oligolepis*; 10—*Moenkhausia celibela*; 11—*Moenkhausia cotinho*; 12—*Hemigrammus* aff. *colletii*; 13—*Moenkhausia* cf. *lepidura*; 14—*Serrapinnus* cf. *notomelas*.; 15—*Aphyocharax* sp.; 16—*Hemigrammus levis*; 17—*Chilodus punctatus*; 18—*Characidium* sp.; 19—*Boulengerella maculata;* 20—*Pseudanos gracilis*; 21—*Curimatopsis cryptica*; 22—*Curimatopsis macrolepis*; 23—*Moenkhausia ceros*; 24—*Hemiodus atranalis*; 25—*Apareiodon* sp.; 26—*Copella callolepis*; 27—*Nannostomus* cf. *beckfordi*; 28—*Nannostomus* sp.; 29—*Nannostomus eques*; 30—*Nannostomus unifasciatus*; 31—*Apistogramma* sp.; 32—*Mesonauta* cf. *festivus*; 33—*Mesonauta insignis*; 34—*Astronotus crassipinnis*; 35—*Acarichthys heckellii;* 36—*Chaetobranchus flavescens*; 37—*Heros efasciatus;* 38—*Hypselecara temporalis;* 39—*Acaronia nassa;* 40 –*Satanoperca acuticeps;* 41—*Cichlasoma amazonarum*; 42—*Caquetaia spectabilis;* 43—*Laetacara flamannellus;* 44—*Astrodoras asterifrons*; 45—*Hoplisoma* cf. *melanistium;* 46—*Peckoltia* cf. *vittata*.; 47—*Ancistrus* sp.

The Characiformes was the most speciose order, with 31 species, followed by the Cichliformes, with 14 species, and the Siluriformes, with six. The most diverse families were the Acestrorhamphidae, with 16 species, and the Cichlidae, with 14 species, followed by the Lebiasinidae, with five species (Table 1; Fig 3).

**Table 1:** Ornamental fish species collected from the three sampling points on the lower Amazon River. P1 = Paracari Stream, P2 = Pitiú Lake, P3 = Açu da Fazenda Stream.

ORDER	Species collected from sampling point
Family	
*Species*	
**CHARACIFORMES**	
**Acestrorhamphidae**	
*Astyanax* aff. *argymarginatus* Garutti, 1999	P3
*Astyanax bimaculatus* (Linnaeus, 1758)	P3
*Bario oligolepis* (Günther, 1864)	P3
*Ctenobrycon spilurus* (Valenciennes, 1850)	P3
*Hemigrammus* aff. *collettii* (Steindachner, 1882)	P2
*Hemigrammus analis* Durbin, 1909	P1
*Hemigrammus durbinae* Ota, Lima & Pavanelli, 2015	P2
*Hemigrammus levis* Durbin, 1908	P1
*Hemigrammus* sp.	P2
*Hemigrammus stictus* (Durbin, 1909)	P1
*Makunaima guianensis* (Eigenmann, 1909)	P3
*Megalamphodus* cf. *bentosi* (Durbin, 1908)	P1
*Moenkhausia celibela* Marinho & Langeani, 2010	P2 and P3
*Moenkhausia ceros* Eigenmann, 1908	P1
*Moenkhausia cotinho* Eigenmann, 1908	P3
*Moenkhausia* cf. *lepidura* (Kner, 1858)	P1
**Anostomidae**	
*Pseudanos gracilis* (Kner, 1858)	P3
**Characidae**	
*Aphyocharax* sp.	P3
*Serrapinnus* cf. *notomelas* (Eigenmann, 1915)	P3
**Chilodontidae**	
*Chilodus punctatus* Müller & Troschel, 1844	P3
**Crenuchidae**	
*Characidium* cf. *zebra* Eigenmann, 1909	P3
**Ctenoluciidae**	
*Boulengerella maculata* (Valenciennes, 1850)	P1
**Curimatidae**	
*Curimatopsis cryptica* Vari, 1982	P1
*Curimatopsis macrolepis* Steindachner, 1876	P2
**Hemiodontidae**	
*Hemiodus atranalis* (Fowler, 1940)	P1
**Lebiasinidae**	
*Copella callolepis* (Regan, 1912)	P1
*Nannostomus* cf. *beckfordi* Günther, 1872	P2
*Nannostomus eques* Steindachner, 1876	P1
*Nannostomus unifasciatus* Steindachner, 1876	P1
*Nannostomus* sp.	P1
**Parodontidae**	
*Apareiodon* sp.	P3
**CICHLIFORMES**	
**Cichlidae**	
*Acarichthys heckelii* (Müller & Troschel, 1849)	P1 and P2
*Acaronia nassa* (Heckel, 1840)	P1
*Apistogramma* sp.	P1
*Astronotus* cf. *crassipinnis* (Heckel, 1840)	P2
*Caquetaia spectabilis* (Steindachner, 1875)	P3
*Chaetobranchus flavescens* Heckel, 1840	P1
*Cichlasoma amazonarum* Kullander, 1983	P2
*Heros efasciatus* Heckel, 1840	P2
*Hypselecara* cf. *coryphaenoides* (Heckel, 1840)	P2
*Hypselecara temporalis* (Günther, 1862)	P2
*Laetacara flamannellus* Ottoni, Bragança, Amorim & Gama, 2012	P1
*Mesonauta* cf. *festivus* (Heckel, 1840)	P1
*Mesonauta insignis* (Heckel, 1840)	P2
*Satanoperca acuticeps* (Heckel, 1840)	P1
**SILURIFORMES**	
**Callichthyidae**	
*Hoplisoma* cf. *melanistium* (Regan, 1912)	P3
*Hoplisoma* sp.	P3
**Doradidae**	
*Astrodoras asterifrons* (Kner, 1853)	P1
*Astrodoras* cf. *asterifrons* (Kner, 1853)	P1
**Loricariidae**	
*Ancistrus* sp.	P3
*Peckoltia* cf. *vittata* (Steindachner, 1881)	P3

It was possible to identify 31 of the 51 species discriminated during the present study, with seven othen being identified to genus. For the remaining 13 taxa it was not possible to obtain a species identification. All identifications were based on the barcode sequence of the MT-CO1 gene in conjunction with the analysis of morphological characteristics by taxomists. Among the species identified, only 19 had the morphological identification corresponding to the molecular identification, based on MT-CO1 sequence deposits in public repositories (see S1 Table). None of the species was found in all of the three sampling. *Acarichthys heckellii* and *Moenkhausia celibela* were the species with the greatest distribution, occurring in two of the three sampled points, Paracari Stream (P1) and Pitiú Lake (P2) and Açu da Fazenda Stream (P3) and Pitiú Lake (P2), respectively.

### Ornamental fish community of Paracari Stream

**Fig 4:**
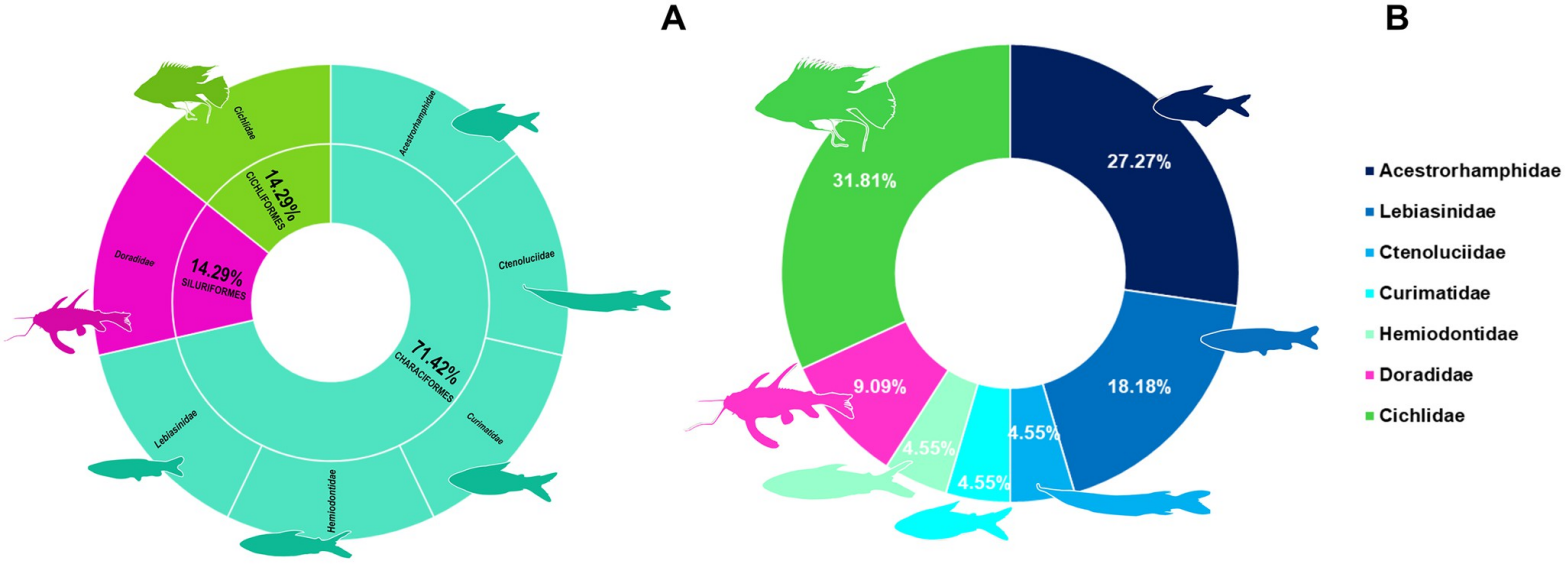
Charts of the diversity of the ornamental fish fauna recorded at Paracari Stream (point 1). **A.** The families recorded per order. **B.** The proportion of species belonging to each family. The silhouettes are representative of the morphology of each family.

The Paracari Stream had a total of 22 fish species, representing seven families and three orders. The most speciose family was the Cichlidae (seven species), followed by the Acestrorhamphidae (six species), and the Lebiasinidae (four species) (Fig 4).

**Fig 5:**
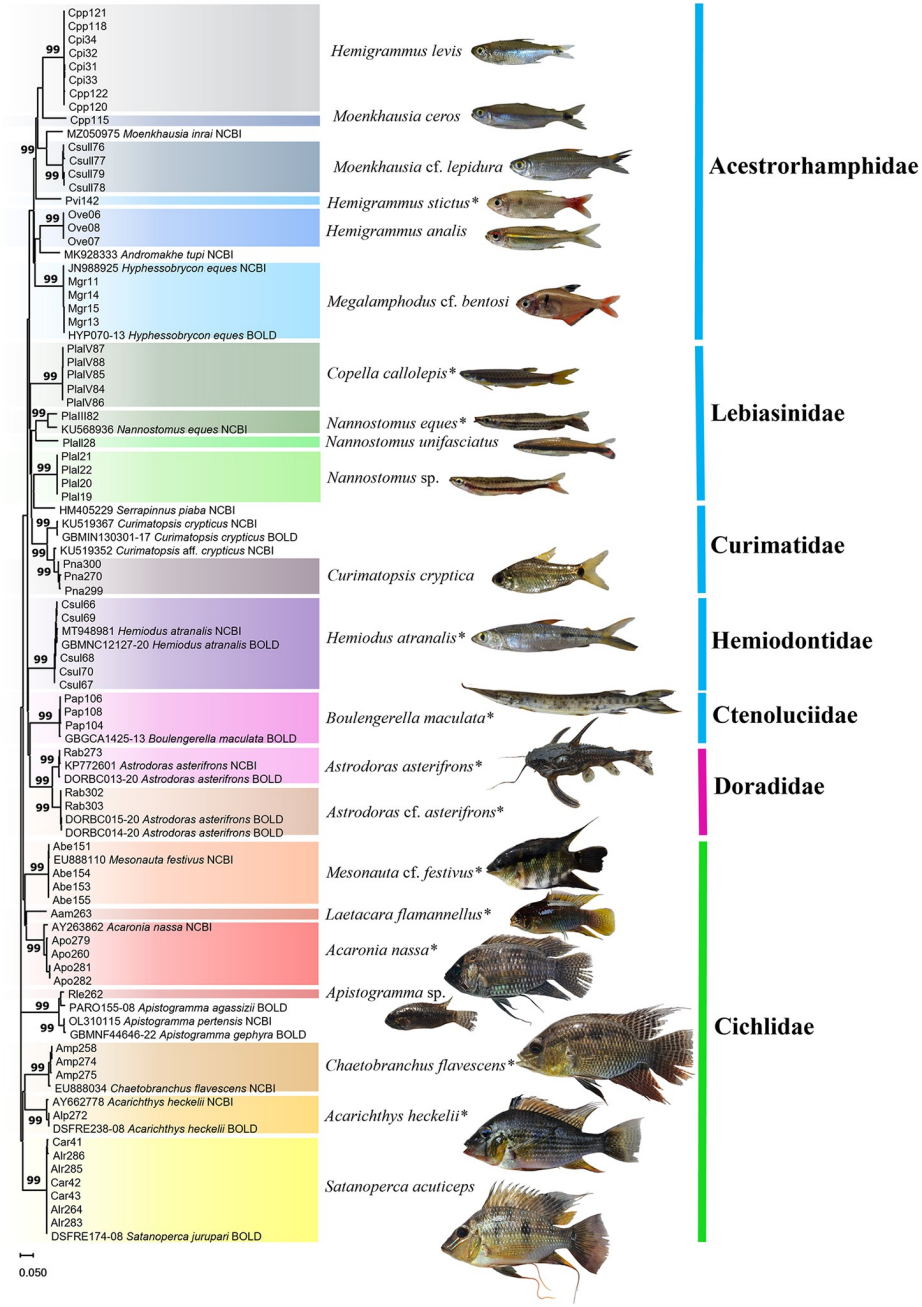
Neighbor-Joining tree (NJ) with individuals from Paracari Stream. NJ tree of the DNA barcode sequences of the fish specimens collected from Paracari Stream, in Monte Alegre, Pará (Brazil), and the reference sequences obtained from the GenBank and BOLD databases. The species (center) are shaded in contrasting colors in the tree (to the left), while the families are color-coded by order (to the right): blue = Characiformes, green = Cichliformes, pink = Siluriformes. The numbers above the nodes indicate the statistical support of the respective branches. US = Unidentified Species. *Species in which molecular identification was consistent with morphological identification.

It was possible to identify the vast majority of the specimens collected from this point to species (Fig 5), with the exception of *Nannostomus* sp. and *Apistogramma* sp.

### Ornamental fish community of Pitiú Lake

**Fig 6:**
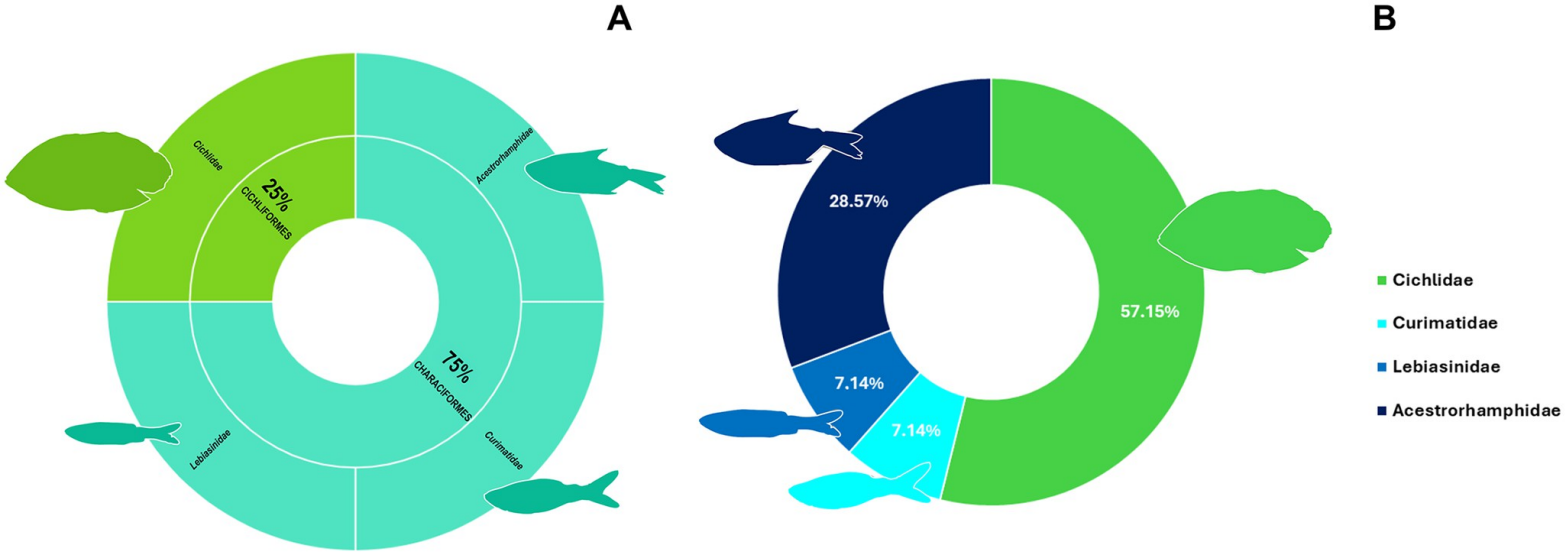
Charts of the diversity of the ornamental fish fauna recorded at Pitiú Lake (point 2). **A.** The families recorded per order. **B.** The proportion of species belonging to each family. The silhouettes are representative of the morphology of each family.

A total of 13 species were collected at this point, representing two orders and four families. The Cichlidae was the most diverse, with seven species, followed by the Acestrorhamphidae, with four (Fig 6).

**Fig 7:**
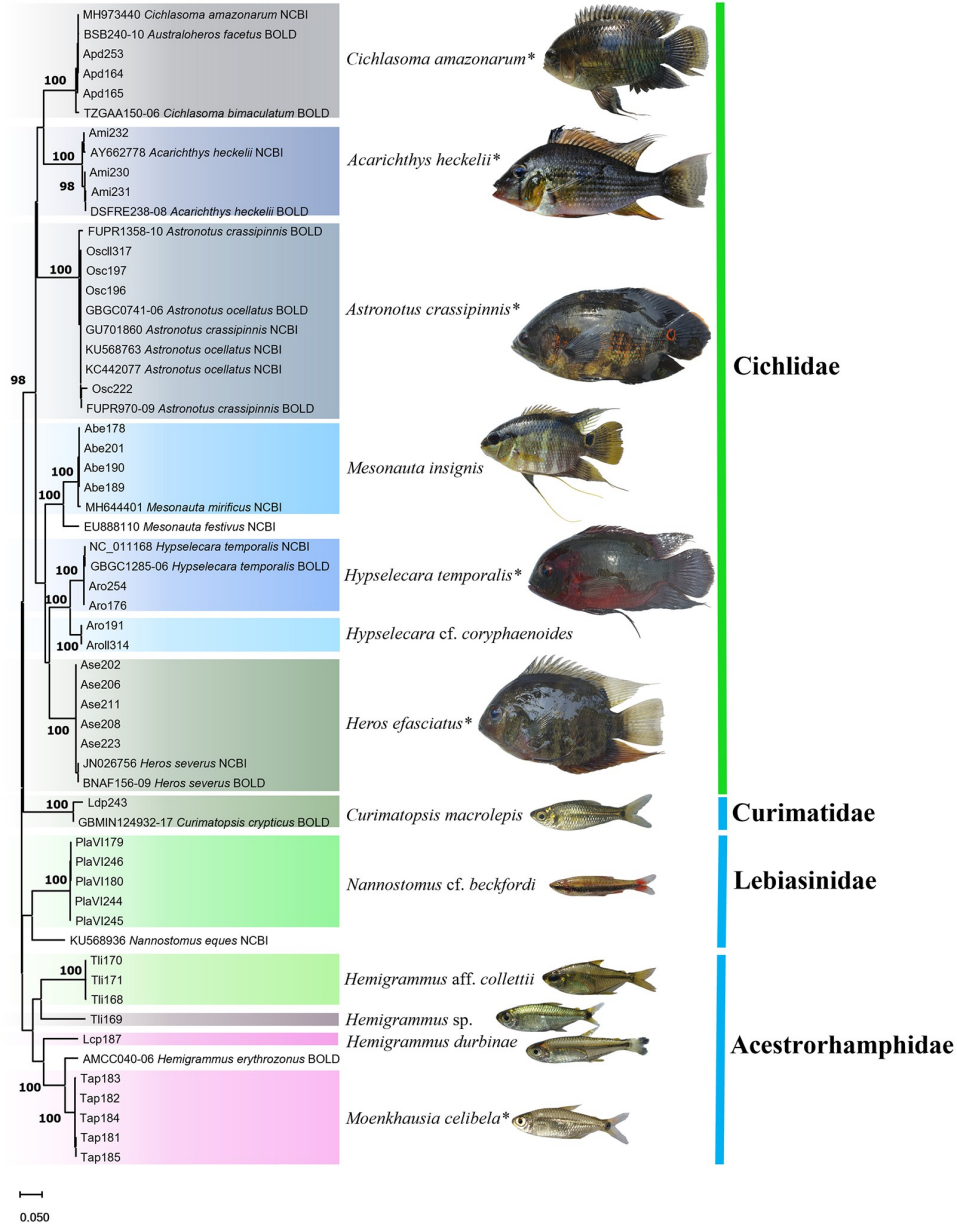
Neighbor-Joining tree (NJ) with individuals from Pitiú Lake. NJ tree of the DNA barcode sequences of the fish specimens collected from Pitiú Lake, in Monte Alegre, Pará (Brazil), and the reference sequences obtained from the public databases. The species (center) are shaded in contrasting colors in the tree (to the left), while the families are color-coded by order (to the right): blue = Characiformes, green = Cichliformes. The numbers above the nodes indicate the statistical support of the respective branches. *Species in which molecular identification was consistent with morphological identification.

All specimens were identified to the lowest possible taxonomic level, with the exception of *Hemigrammus* sp. (Fig 7).

### Ornamental fish community of Açu da Fazenda Stream

**Fig 8:**
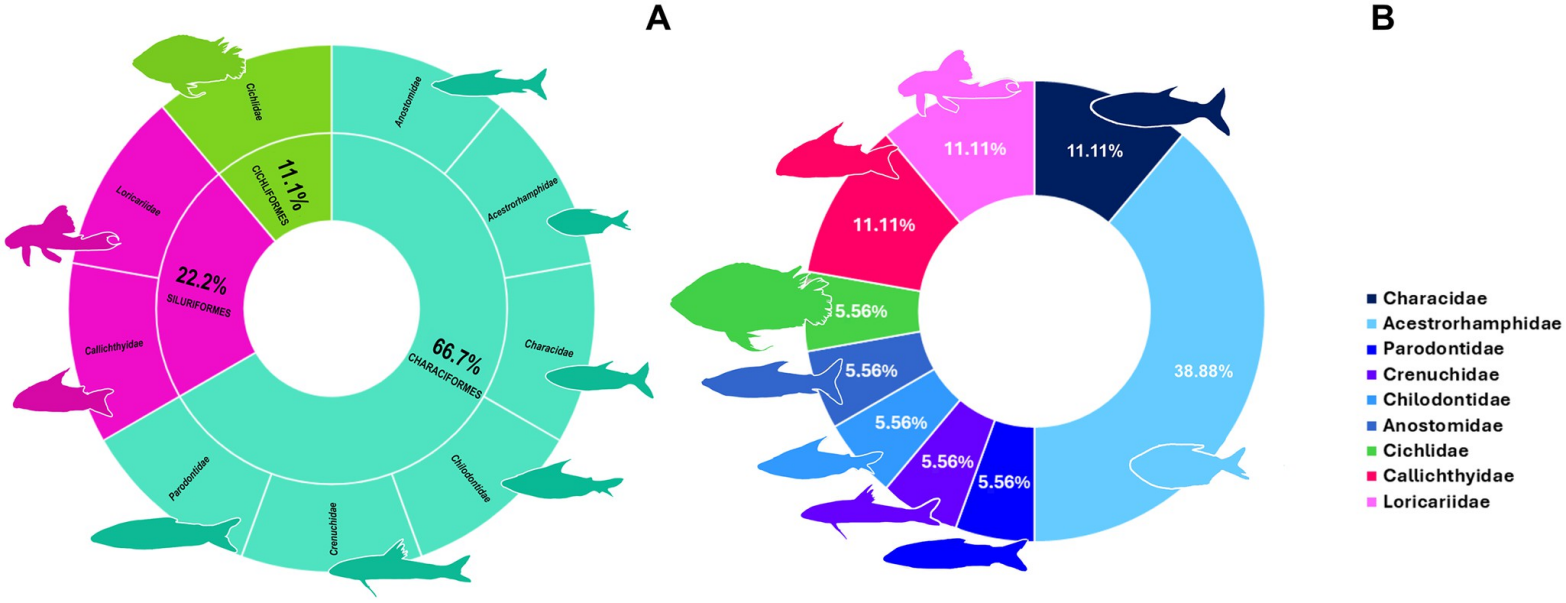
Charts of the diversity of the ornamental fish fauna recorded at Açu da Fazenda Stream (point 3). **A.** The families recorded per order. **B.** The proportion of species belonging to each family. The silhouettes are representative of the morphology of each family.

**Fig 9:**
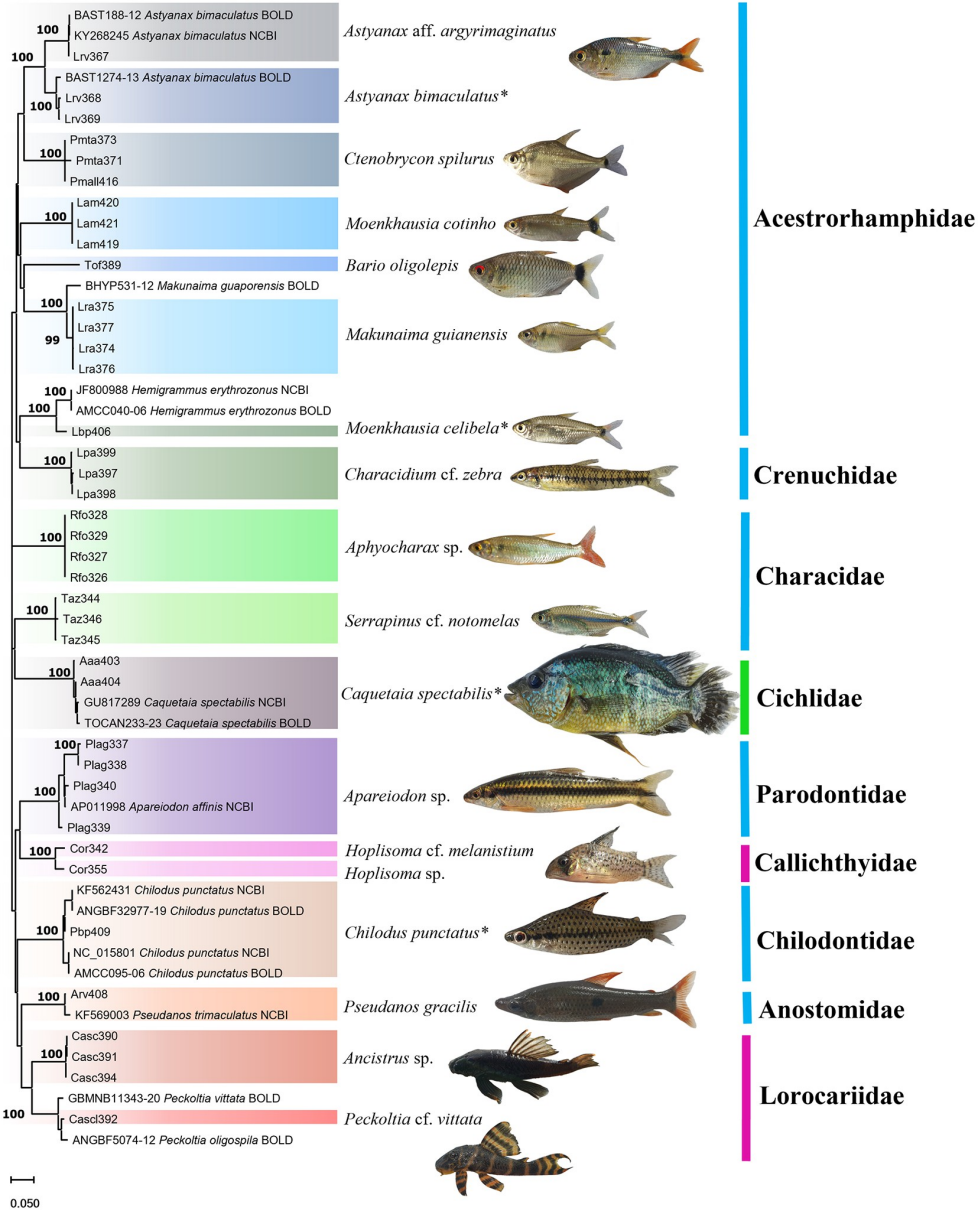
Neighbor-Joining tree (NJ) with individuals from Açu da Fazenda Stream. NJ tree of the DNA barcode sequences of the fish specimens collected from Açu da Fazenda Stream, in Monte Alegre, Pará (Brazil), and the reference sequences obtained from the GenBank and BOLD databases. The species (center) are shaded in contrasting colors in the tree (to the left), while the families are color-coded by order (to the right): blue = Characiformes, green = Cichliformes, pink = Siluriformes. The numbers above the nodes indicate the statistical support of the respective branches. *Species in which molecular identification was consistent with morphological identification.

A total of 18 species were collected from point 3, representing three orders and nine families (Fig 8). The Acestrorhamphidae was the most speciose family, with seven species, followed by the Characidae, Callichthyidae and Loricariidae, each with two species. It was possible to identify all but four of the taxa to species level (Fig 9).

## Discussion

The habitats surveyed here presented a considerable diversity of fish, with the majority of the species being collected at only one of the sampling points. This may indicate that the distribution of these species is being influenced primarily by the geomorphological factors of the drainage network and specific ecological characteristics, given that the three sites have distinct landscapes [39, 40], which are dominated by complex habitats appropriate for an ample variety of species.

The composition of the fish fauna was broadly similar to those reported by previous studies of the freshwater fish fauna of aquatic Amazonian ecosystems, with a predominance of Characiformes. In the Amazon basin, Characiformes is the richest order with more than 1060 valid species, distributed in a variety of freshwater environments, followed by Siluriformes with approximately 950 and Cichliformes with approximately 260 species [2]. However, similar to this study, in which Cichliformes had more species than Siluriformes, other faunistic surveys in the Amazon basin revealed an inversion in the number of Cichliformes and Siluriformes species, mainly in lentic environments [41].

On the other hand, the relatively low diversity of Siluriformes recorded in the present study may be related to the nocturnal and benthic habits of many of its representatives which would tend to reduce their probability of being captured by the types of fishing gear employed [41]. As an example, the members of Pimelodidae and Auchenipteridae, which despite being diverse in the region, were not collected in the present study.

### New records of occurrence for the lower Amazon and undescribed species

One of the species collected from point 1 –*Laetacara flamannelus*–was recorded in the study region for the first time, given that all previous records were obtained from the rivers of the neighboring state of Amapá. This species has been considered to be conspecific to *Laetacara curviceps* by some authors, given their marked morphological similarities [42], which hampers the reliable identification of specimens and has led to the use of molecular tools in other studies, to provide a more precise identification [43, 44]. These records of new occurrences are fundamentally important, in particular in the context of the major hydrographic systems in the Amazon basin and other regions of Brazil, which have a highly diverse freshwater fish fauna. Reliable data on the occurrence of the different species are essential for understanding the similarities and differences between these distinct environments [45].

The drainages in the state of Pará, including the lower Amazon, the area where the sampled points are located, present an underestimated ichthyofauna, with the presence of several undescribed species [46, 47]. In addition to the occurrence record, from the joint identification between morphological and molecular tools it was possible to record individuals whose species have not yet been formally described, such as *Hemigrammus* aff. *collettii*, *Astyanax* aff. *argyrimarginatus*, *Apareiodon* sp. and *Aphyocarax* sp.

New fish species have been described relatively frequently in recent years. According to [48], 100 new species of fish are discovered annually, on average, in South America. In fact, a large number of recent publications have recorded new fish species from the Amazon basin, including the floodplain of the lower Amazon [47, 49–54].

### Identification of the species

The reliable identification of species is fundamental to the inventory and comparison of the fish faunas of distinct environments [55, 56], as well as the development of captive breeding programs for the most important species. The reliable identification of species is essential for the definition of their ecological, behavioral, and evolutionary characteristics, as well as other diagnostic traits of the taxon [57], which are basic parameters in most biological disciplines [58].

On the other hand, the incorrect identification of a species can have a series of potentially negative consequences, in particular, for the conservation and management of its populations in the wild. One recent example is the case of a *Corydoras* species from the Tapajós River basin, which was being traded on the ornamental fish market before even being formally described [59]. Situations like this may have an impact on the conservation of a species, given that, in many cases, the fish are not bred in captivity, but are extracted from the wild for sale. Without the accurate indication of the species being extracted from the wild, it is impossible to determine whether their populations are threatened by the trade. This further highlights the need for the effective understanding of the taxonomic composition of the fish fauna of any given region.

In this study, of the 51 taxa discriminated, it was possible to identify more than 62% of the specimens to the species level, which was equivalent to 31 species, in addition to seven taxa that were identified to the genus level. The remaining taxa were species that had not yet been formally described or species whose identification we were uncertain about, and for these we could only indicate the most closely related species.

Despite the care taken to identify accurately the fish species sampled in the present study, some limitations did arise, such as the reduced availability of specimens in the public databases, in some cases, and the mislabeling of sequences, which impeded the successful recognition of some individuals, as in the case of specimen PMTA 373 (see S1 Table). The comparison with the GenBank sequences indicated that the species could be either *Astyanax bimaculatus* or *Psellogrammus kennedyi*, whereas the BOLD sequences included three other possibilities in addition to these two species–*Ctenobrycon hauxwellianus*, *Ctenobrycon* sp., and *Astyanax* sp. This impasse was resolved by the morphological diagnosis, however, which revealed that the species was in fact *Ctenobrycon spilurus* (S1 Table).

One other example was specimen PIV 142 (see S1 Table), whose MT-CO1 gene sequence was 99.49% similar to that of *Hyphessobrycon* sp. from the public databases and 99.30% similar to that of *Hemigrammus stictus*, although the specimen was assigned to the latter species, based on its phenotype. Similarly, while the comparisons of the MT-CO1 gene sequences identified five possible species for specimen APD 164 (see S1 Table)–*Aequidens tetramerus*, *Australoheros facetus*, *Cichlasoma amazonarum*, *Cichlasoma orientale*, and *Cichlasoma bimaculatum*–the morphological analysis identified *C*. *amazonarum*. It seems likely that these contradictions are derived primarily from the mislabeling of the DNA sequences deposited in the public databases, a problem that has been identified in a number of previous studies [60].

Other inconsistencies were noted, mainly in cases where differences in morphology are not so evident, such as for species of the genera *Astronotus*, *Moenkhausia*, *Heros* and *Mesonauta*, showing a high similarity (>98%) for two species when consulted in the public repositories. This further reinforces the value of an integrated approach, which combines molecular and morphological data to provide the most reliable possible identification of the specimens [61]. The integrated approach is a powerful tool for the identification of taxa, in particular when the study fauna is megadiverse, as in the case of the streams of the Amazon basin. Despite the potential of this approach, relatively few studies of the fish fauna of Amazonian streams have integrated molecular and morphological data up to now [60].

One other factor that needs to be considered here is the application of the 2% divergence threshold as the standard for species identification using the DNA barcode. This threshold was established by [30], who originally suggested using the 5’ portion of the MT-CO1 gene as a barcode for the identification of metazoan species [62]. Even so, the barcode of the MT-CO1 gene is clearly a valuable tool, even for the identification of cryptic species [63]. A number of recent studies have revealed new fish diversity in the Amazon basin, such as that of [64], who described a new species of the genus *Hyphessobrycon* from the region of the lower Tapajós River based on the portion of the MT-CO1 gene used as DNA barcode. This species–*Hyphessobrycon cantoi*–is one of the first truly cryptic species described from the Amazon basin.

The results of the present study highlight the diversity of species with potential for the ornamental fish trade in the streams and floodplain lakes of the lower Amazon River, as well as corroborate the value of the integrated approach of molecular and morphological identification as a diagnostic tool for reliable identification of taxa. A species of *Laetacara* was recorded for the first time in the Lower Amazon region, and evidence was found for the possible existence of species that have not yet been formally described of *Aphyocharax*, *Astyanax*, *Apareiodon* and *Hemigrammus*.

## Supporting information

S1 Table Molecular and morphological identification of the specimens collected in the present study. P1 = Paracari Stream; P2 = Pitiú Lake; P3 = Açu da Fazenda Stream. GenBank = National Center for Biotechnology Information (NCBI). BOLD = Barcode of Life Data System.
(PDF)
