# Supplementary material for: Integrative use of DNA barcode and morphology reveal high level of diversity in the ornamental fish on the lower Amazon basin
Source: PLoS One. 2024 Dec 30;19(12):e0316455. doi: 10.1371/journal.pone.0316455 (PMC11684679; doi:10.1371/journal.pone.0316455)
Supplement: S1 Table — P1 = Paracari Stream; P2 = Pitiú Lake; P3 = Açu da Fazenda Stream. GenBank = National Center for Biotechnology Information (NCBI). BOLD = Barcode of Life Data System. (PDF) [file pone.0316455.s001.pdf]

S1 Table

Catalog Number	Point	GenBank (% similarity)	Accession code	BOLD (% similarity)	Accession code	Morphological ID
AAM 263	P1	*Laetacara dorsigera* 93,70 %	MH449587	*Laetacara flamannellus* 99,83 %	PRIVATE	*Laetacara flamannellus*
ALP 272	P1	*Acarichthys heckelii* 100 %	AY662778	*Acarichthys heckelii* 100%	DSFRE238-08	*Acarichthys heckelii*
ALR264	P1	*Satanoperca* sp. 100 %	MH931745	*Satanoperca jurupari* 100%	DSFRE174-08	*Satanoperca acuticeps*
ALR283	P1	*Satanoperca* sp. 100 %	MH931745	*Satanoperca jurupari* 100%	DSFRE174-08	
ALR 285	P1	*Satanoperca* sp. 100 %	MH931745	*Satanoperca jurupari* 99,83%	DSFRE174-08	
ALR 286	P1	*Satanoperca* sp. 100 %	MH931745	*Satanoperca jurupari* 98,78%	DSFRE174-08	
AMP 258	P1	*Chaetobranchus flavescens* 98,46 %	EU888034	*Chaetobranchus flavescens* 98,64%	PRIVATE	*Chaetobranchus flavescens*
AMP 274	P1	*Chaetobranchus flavescens* 98,46 %	EU888034	*Chaetobranchus flavescens* 98,64%	PRIVATE	
AMP 275	P1	*Chaetobranchus flavescens* 98,12 %	EU888034	*Chaetobranchus flavescens* 98,13 %	PRIVATE	
APO 260	P1	*Acaronia nassa* 97,26 %	AY263862	*Acaronia nassa* 98,30 %	PRIVATE	*Acaronia nassa*
APO 279	P1	*Acaronia nassa* 96,41 %	AY263862	*Acaronia nassa* 97,95%	PRIVATE	
APO 281	P1	*Acaronia nassa* 97,38 %	AY263862	*Acaronia nassa* 97,45%	PRIVATE	
APO 282	P1	*Acaronia nassa* 97,38 %	AY263862	*Acaronia nassa* 97,45%	PRIVATE	
CAR 41	P1	*Satanoperca* sp. 100%	MH931745	*Satanoperca jurupari* 99,65%	DSFRE174-08	*Satanoperca acuticeps*

Catalog Number	Point	GenBank (% similarity)	Accession code	BOLD (% similarity)	Accession code	Morphological ID
CAR 42	P1	*Satanoperca* sp. 100 %	MH931745	*Satanoperca jurupari* 100%	DSFRE174-08	
CAR 43	P1	*Satanoperca* sp. 100 %	MH931745	*Satanoperca jurupari* 100%	DSFRE174-08	
CPI 31	P1	*Hemigrammus* sp.	OR732933	*Moenkausia comma* 99,33%,<br>*Moenkhausia* cf. *intermedia* 99,66%,<br>*Parapristella aubynei* 99,15%	PRIVATE	*Hemigrammus levis*
CPI 32	P1	*Hemigrammus* sp. 96,12%	OR732933	*Moenkausia comma* 99,49%,<br>*Moenkausia* cf. *intermedia* 99,15%,<br>*Parapristella aubynei* 98,64%	PRIVATE<br>PRIVATE	
CPI 33	P1	*Hemigrammus* sp. 96,30%	OR732933	*Moenkausia comma* 100%,<br>*Moenkausia* cf. *intermedia* 99,66%,<br>*Parapristella aubynei* 99,15%	PRIVATE	
CPI 34	P1	*Hemigrammus* sp. 96,49%	OR732933	*Moenkausia comma* 99,83%,<br>*Moenkausia cf. intermedia* 99,49%,<br>*Parapristella aubynei* 98,98%	PRIVATE	
CSUI66	P1	*Hemiodus atranalis* 99,65%	MT948981	*Hemiodus atranalis* 99,82%	GBMNC12125-20	*Hemiodus atranalis*
CSUI67	P1	*Hemiodus atranalis* 99,12%	MT948981	*Hemiodus atranalis* 99,65%	GBMNC12127-20	
CSUI68	P1	*Hemiodus atranalis* 99,47%	MT948981	*Hemiodus atranalis* 99,63%	GBMNC12125-20	
CSUI69	P1	*Hemiodus atranalis* 99,47%	MT948981	*Hemiodus atranalis* 99,63%	GBMNC12125-20	
CSUI70	P1	*Hemiodus atranalis* 99,63%	MT948981	*Hemiodus atranalis* 99,81%	GBMNC12125-20	

Catalog Number	Point	GenBank (% similarity)	Accession code	BOLD (% similarity)	Accession code	Morphological ID
CSUII76	P1	*Moenkhausia inrai* 88,26%	MZ050975	-	-	*Moenkhausia* cf. *lepidura*
CSUII77	P1	*Moenkhausia inrai* 88,61%	MZ050975	-	-	
CSUII78	P1	*Moenkhausia inrai* 88,76%	MZ050975	-	-	
CSUII79	P1	*Moenkhausia inrai* 88,59%	MZ050975	-	-	
MGR 11	P1	*Hyphessobrycon eques* 99,83%	KU568867	*Hyphessobrycon eques* 100%	HYP070-13	*Megalamphodus* cf. *bentosi*
MGR 13	P1	*Hyphessobrycon eques* 99,83%	KU568867	*Hyphessobrycon eques* 100%	HYP070-13	
MGR 14	P1	*Hyphessobrycon eques* 99,83%	KU568867	*Hyphessobrycon eques* 100%	HYP070-13	
MGR 15	P1	*Hyphessobrycon eques* 99,83%	KU568867	*Hyphessobrycon eques* 100%	HYP070-13	
OVE 06	P1	*Andromakhe tupi* 84,81 %	MK928333	-	-	*Hemigrammus analis*
OVE 07	P1	*Andromakhe tupi* 84,97 %	MK928333	-	-	
OVE 08	P1	*Andromakhe tupi* 84,97 %	MK928333	-	-	
PAP 104	P1	*Boulengerella maculata* 99,16 %	AB070207	*Boulengerella maculata* 98,99 %	GBGCA1425-13	*Boulengerella maculata*
PAP 106	P1	*Boulengerella maculata* 99,33 %	AB070207	*Boulengerella maculata* 99,17 %	GBGCA1425-13	
PAP 108	P1	*Boulengerella maculata* 99,33 %	AB070207	*Boulengerella maculata* 99,17 %	GBGCA1425-13	

Catalog Number	Point	GenBank (% similarity)	Accession code	BOLD (% similarity)	Accession code	Morphological ID
PLA I19	P1	*Labeo boggut* 85,21 %	MK993538	-	-	*Nannostomus* sp.
PLA I20	P1	*Labeo boggut* 85,21 %	MK993538	-	-	
PLA I21	P1	*Labeo boggut* 85,21 %	MK993538	-	-	
PLA I22	P1	*Labeo boggut* 85,21 %	MK993538	-	-	
PLAII28	P1	*Nannostomus eques* 85,62 %	KU568936	-	-	*Nannostomus unifasciatus*
PLAIII82	P1	*Nannostomus eques* 93,16 %	KU568936	*Nannostomus eques* 99,49%	PRIVATE	*Nannostomus eques*
PLAIV84	P1	*Chandramara chandramara* 83,33%	KT762367	*Copella callolepis* 99,66%	PRIVATE	*Copella callolepis*
PLAIV85	P1	*Chandramara chandramara* 83,33%	KT762367	*Copella callolepis* 99,66%	PRIVATE	
PLAIV86	P1	*Chandramara chandramara* 83,16%	KT762367	*Copella callolepis* 99,50%	PRIVATE	
PLAIV87	P1	*Chandramara chandramara* 83,33%	KT762367	*Copella callolepis* 99,66%	PRIVATE	
PLAIV88	P1	*Chandramara chandramara* 83,33%	KT762367	*Copella callolepis* 99,66%	PRIVATE	
PIV 142	P1	*Astyanax mexicanus* 85,37%	HM379754	*Hyphessobrycon* sp. 99,49 %<br>*Hemigrammus stictus* 99,30%	PRIVATE	*Hemigrammus stictus*
RAB 273	P1	*Astrodoras asterifrons* 99,64 %	KP772601	*Astrodoras asterifrons* 99,32 %	DORBC013-20	*Astrodoras asterifrons*
RAB 302	P1	*Astrodoras asterifrons* 94,29 %	KP772601	*Astrodoras asterifrons* 100 %	DORBC014-20	
RAB 303	P1	*Astrodoras asterifrons* 94,29 %	KP772601	*Astrodoras asterifrons* 100 %	DORBC014-20	

Catalog Number	Point	GenBank (% similarity)	Accession code	BOLD (% similarity)	Accession code	Morphological ID
RLE 262	P1	*Apistogramma pertensis* 95,83 %	OL310115	*Apistogramma agassizii* 97,09 %	PARO155-08	*Apistogramma* sp.
ABE 151	P1	*Mesonauta festivus* 99,81%	EU888110	*Mesonauta festivus* 97,61%	PRIVATE	*Mesonauta* cf. *festivus*
ABE 153	P1	*Mesonauta festivus* 99,81%	EU888110	*Mesonauta festivus* 97,78%	PRIVATE	
ABE 154	P1	*Mesonauta festivus* 99,81%	EU888110	*Mesonauta festivus* 97,78%	PRIVATE	
ABE 155	P1	*Mesonauta festivus* 99,81%	EU888110	*Mesonauta festivus* 97,61%	PRIVATE	
PNA270	P1	*Curimatopsis* aff. *crypticus* 96,93%	KU519352	-	-	*Curimatopsis cryptica*
PNA299	P1	*Curimatopsis* aff. *crypticus* 97,10%	KU519352	*Curimatopsis crypticus* 97,09%	PRIVATE	
PNA300	P1	*Curimatopsis* aff. *crypticus* 97,61%	KU519352	*Curimatopsis crypticus* 97,61 %	PRIVATE	
CPP 118	P1	*Hemigrammus* sp. 96,49%	OR732933	*Moenkausia comma* 99,83%, *Moenkausia* cf. *intermedia* 99,49%, *Parapristella aubynei* 98,98%	PRIVATE	
CPP 120	P1	*Hemigrammus* sp. 95,93%	OR732933	*Moenkausia comma* 99,66%, *Moenkausia* cf. *intermedia* 99,32%, *Parapristella aubynei* 98,81%	PRIVATE	
CPP121	P1	*Hemigrammus* sp. 96,49%	OR732933	*Moenkausia comma* 99,83%, *Moenkausia* cf. *intermedia* 99,49%, *Parapristella aubynei* 98,98%	PRIVATE	
CPP122	P1	*Hemigrammus* sp. 96,29%	OR732933	*Moenkausia comma* 99,32%, *Moenkausia* cf. *intermedia* 99,49%, *Parapristella aubynei* 98,80%	PRIVATE	

Catalog Number	Point	GenBank (% similarity)	Accession code	BOLD (% similarity)	Accession code	Morphological ID
CPP115	P1	*Hemigrammus levis* 97,60%	OR732934	*Hemigrammus levis* 97,77%	TOCAN356-23	*Moenkhausia ceros*
ABE 178	P2	*Mesonauta mirificus* 99,01%	MH644401	*Mesonauta mirificus* 99,13%<br>*Mesonauta guyanae* 98,63%	PRIVATE<br>PRIVATE	*Mesonauta insignis*
ABE 189	P2	*Mesonauta mirificus* 99,21%	MH644401	*Mesonauta mirificus* 99,30%<br>*Mesonauta guyanae* 98,80%	PRIVATE<br>PRIVATE	
ABE 190	P2	*Mesonauta mirificus* 99,21%	MH644401	*Mesonauta mirificus* 99,30%<br>*Mesonauta guyanae* 98,80%	PRIVATE<br>PRIVATE	
ABE 201	P2	*Mesonauta mirificus* 99,21%	MH644401	*Mesonauta mirificus* 99,30%<br>*Mesonauta guyanae* 98,80%	PRIVATE<br>PRIVATE	
AMI 230	P2	*Acarichthys heckelii* 98,35 %	AY662778	*Acarichthys heckelii* 100%	DSFRE238-08	*Acarichthys heckelii*
AMI 231	P2	*Acarichthys heckelii* 98,35 %	AY662778	*Acarichthys heckelii* 100%	DSFRE238-08	
AMI 232	P2	*Acarichthys heckelii* 99,45 %	AY662778	*Acarichthys heckelii* 98,95 %	DSFRE238-08	
APD 164	P2	*Australoheros facetus* 100%<br>*Cichlasoma amazonarum* 100%<br>*Cichlasoma orientale* 98,34%	HM405091<br>MH973440<br>KF938517	*Australoheros facetus* 100%<br>*Cichlasoma amazonarum* 100%<br>*Aequidens tetramerus* 100%<br>*Cichlasoma bimaculatum* 98,80%<br>*Cichlasoma orientale* 98,29%	BSB240-10<br>PRIVATE<br>PRIVATE<br>PRIVATE<br>RENA010-16	*Cichlasoma amazonarum*

Catalog Number	Point	GenBank (% similarity)	Accession code	BOLD (% similarity)	Accession code	Morphological ID
APD 165	P2	*Australoheros facetus* 99,65%<br>*Cichlasoma amazonarum* 99,58%	HM405091<br>MH973440	*Australoheros facetus* 99,65%<br>*Cichlasoma amazonarum* 99,65%<br>*Aequidens tetramerus* 99,65%<br>*Cichlasoma bimaculatum* 98,46%<br>*Cichlasoma orientale* 98,12%	BSB240-10<br>PRIVATE<br>PRIVATE<br>PRIVATE<br>PRIVATE	
APD 253	P2	*Australoheros facetus* 100%<br>*Cichlasoma amazonarum* 100%<br>*Cichlasoma orientale* 98,34%	HM405091<br>MH973440<br>KF938517	*Australoheros facetus* 100%<br>*Cichlasoma amazonarum* 100%<br>*Aequidens tetramerus* 100%<br>*Cichlasoma bimaculatum* 98,80%<br>*Cichlasoma orientale* 98,29%	BSB240-10<br>PRIVATE<br>PRIVATE<br>PRIVATE<br>RENA010-16	
ASE202	P2	*Heros severus* 99,48%	JN026756	*Heros efasciatus* 99,83%<br>*Heros severus* 99,51%	PRIVATE<br>BNAF156-09	*Heros efasciatus*
ASE206	P2	*Heros severus* 99,48%	JN026756	*Heros efasciatus* 99,83%<br>*Heros severus* 99,51%	PRIVATE<br>BNAF156-09	
ASE208	P2	*Heros severus* 99,48%	JN026756	*Heros efasciatus* 99,83%<br>*Heros severus* 99,51%	PRIVATE<br>BNAF156-09	
ASE 211	P2	*Heros severus* 99,48%	JN026756	*Heros efasciatus* 99,83%<br>*Heros severus* 99,51%	PRIVATE<br>BNAF156-09	
ASE223	P2	*Heros severus* 99,48%	JN026756	*Heros efasciatus* 99,83%<br>*Heros severus* 99,51%	PRIVATE<br>BNAF156-09	
LCP 187	P2	*Hemigrammus marginatus* 86,22 %	JN988891	-	-	*Hemigrammus durbinae*

Catalog Number	Point	GenBank (% similarity)	Accession code	BOLD (% similarity)	Accession code	Morphological ID
OSC 196	P2	*Astronotus ocellatus* 100 %<br>*Astronotus crassipinnis* 100 %	NC-00905<br>GU701860	*Astronotus crassipinnis* 100 %<br>*Astronotus ocellatus* 100 %	FUPR974-09<br>GBGC0741-06	*Astronotus crassipinnis*
OSC 197	P2	*Astronotus ocellatus* 99,83 %<br>*Astronotus crassipinnis* 99,83 %	NC-009058<br>GU701860	*Astronotus ocellatus* 99,83 %<br>*Astronotus crassipinnis* 99,83 %	ANGBF18081-19<br>FUPR974-09	
OSCII317	P2	*Astronotus ocellatus* 99,83 %<br>*Astronotus crassipinnis* 99,83 %	NC-009058<br>KU568763	*Astronotus ocellatus* 99,84 %<br>*Astronotus crassipinnis* 99,84 %	GBGC0741-06<br>FUPR973-09	*Astronotus crassipinnis*
OSC 222	P2	*Astronotus ocellatus* 99,45 %<br>*Astronotus crassipinnis* 99,44 %	NC-009058<br>GU701860	*Astronotus ocellatus* 99,45 %<br>*Astronotus crassipinnis* 99,44 %	IOFBI067-11<br>FUPR974-09	
PLAVI179	P2	*Myxiops aphos* 85,23%	KY327452	*Nannostomus beckfordi* 98,95 %	PRIVATE	*Nannostomus* cf. *beckfordi*
PLAVI180	P2	*Nannostomus eques* 85,51 %	KU568936	*Nannostomus beckfordi* 99,13 %	PRIVATE	
PLAVI244	P2	*Nannostomus eques* 85,51 %	KU568936	*Nannostomus beckfordi* 99,13 %	PRIVATE	
PLAVI245	P2	*Nannostomus eques* 85,69 %	KU568936	*Nannostomus beckfordi* 99,13 %	PRIVATE	
PLAVI246	P2	*Myxiops aphos* 85,23%	KY327452	*Nannostomus beckfordi* 98,95 %	PRIVATE	
TAP 181	P2	*Hemigrammus erythrozonus* 94,99 %	JF800988	*Moenkhausia celibela* 98,78 %	PRIVATE	*Moenkhausia celibela*
TAP 182	P2	*Hemigrammus erythrozonus* 94,82 %	JF800988	*Moenkhausia celibela* 98,95%	PRIVATE	
TAP 183	P2	*Hemigrammus erythrozonus* 94,65 %	JF800988	*Moenkhausia celibela* 99,13%	PRIVATE	
TAP 184	P2	*Hemigrammus erythrozonus* 94,82 %	JF800988	*Moenkhausia celibela* 98,95%	PRIVATE	

Catalog Number	Point	GenBank (% similarity)	Accession code	BOLD (% similarity)	Accession code	Morphological ID
TAP 185	P2	*Hemigrammus erythrozonus* 94,99 %	JF800988	*Moenkhausia celibela* 98,78 %	PRIVATE	
TLI 168	P2	*Moenkhausia collettii* 86,16 %	MZ051443	*Moenkhausia* sp. 97,72%	PRIVATE	*Hemigrammus* aff. *collettii*
TLI 169	P2	*Astyanax mexicanus* 84,86 %	OQ889367	*Hemigrammus lunatus* 100%	PRIVATE	
TLI 170	P2	*Moenkhausia collettii* 86,13 %	MZ051443	*Moenkhausia* sp. 97,57%	PRIVATE	
TLI 171	P2	*Moenkhausia collettii* 86,30 %	MZ051443	*Moenkhausia* sp. 97,74%	PRIVATE	
ARO176	P2	*Hypselacara temporalis* 99,80%	DQ119219	*Hypselacara temporalis* 99,83%	GBMTG1335-16	*Hypselecara temporalis*
ARO254	P2	*Hypselacara temporalis* 99,80%	DQ119219	*Hypselacara temporalis* 99,83%	GBMTG1335-16	
ARO191	P2	*Hypselacara temporalis* 94,24%	NC-011168	*Hypselecara temporalis* 99,15%	PRIVATE	
AROII314	P2	*Hypselacara temporalis* 94,49%	NC-011168	-	-	*Hypselecara* cf. *coryphaenoides*
LDP 243	P2	*Curimatopsis crypticus* 99,13%	KU519366	*Curimatopsis crypticus* 99,13%<br>*Hyphessobrycon* sp. 98,40%	GBMIN130302-17<br>PRIVATE	*Curimatopsis macrolepis*
CASCL 392	P3	*Peckoltia oligospila* 98,05%<br>*Peckoltia vittata* 97,56 %	JF747008<br>MN854567	*Peckoltia oligospila* 98,04 %<br>*Peckoltia capitulata* 97,97%	ANGBF5081-12<br>PRIVATE	*Peckoltia* cf. *vittata*
CASC390	P3	*Ancistrus* sp. 94, 95%	KP960568	*Ancistrus* gr. *leucosticus* 98,66%	PRIVATE	*Ancistrus* sp.
CASC391	P3	*Ancistrus* sp. 94, 95%	KP960568	*Ancistrus* gr. *leucosticus* 98,66%	PRIVATE	
CASC394	P3	*Ancistrus* sp. 95, 11%	KP960568	*Ancistrus* gr.*leucosticus* 98,83%	PRIVATE	

Catalog Number	Point	GenBank (% similarity)	Accession code	BOLD (% similarity)	Accession code	Morphological ID
COR342	P3	*Corydoras duplicareus* 95,77%	NC-049095	*Corydoras schwartzi* 99,83%<br>*Corydoras urucu* 98,67%	PRIVATE<br>EARLY RELEASE	*Hoplisoma* cf. *melanistium*
COR355	P3	*Corydoras duplicareus* 99,84%	NC-049095	*Corydoras duplicareus* 99,83 %<br>*Corydoras kanei* 99,50%<br>*Corydoras bicolor* 99,51%<br>*Corydoras melanistius* 99,51%	PRIVATE<br>HEEN054-20<br>PRIVATE<br>PRIVATE	*Hoplisoma* sp.
LPA 397	P3	*Characidium zebra* 97,06%	MZ051944	*Characidium* aff. *zebra* 97,62%	PRIVATE	*Characidium* cf. *zebra*
LPA 398	P3	*Characidium zebra* 96,74%	MZ051944	*Characidium* aff. *zebra* 97,28%	PRIVATE	
LPA 399	P3	*Characidium zebra* 97,39%	MZ051944	*Characidium* aff. *zebra* 97,62%	PRIVATE	
LAM 419	P3	*Astyanax lineatus* 83,77%	MK928334	-	-	*Moenkhausia cotinho*
LAM 420	P3	*Astyanax lineatus* 83,77%	MK928334	-	-	
LAM 421	P3	*Astyanax lineatus* 83,77%	MK928334	-	-	
LBP 406	P3	*Moenkhausia bonita* 94,27%	OR922609	*Moenkhausia celibela* 98,99 %	PRIVATE	*Moenkhausia celibela*
LRA 374	P3	*Makunaima guaporensis* 96,35 %	KY268181	-	-	*Makunaima guianensis*
LRA 375	P3	*Makunaima guaporensis* 96,35 %	KY268181	-	-	
LRA 376	P3	*Makunaima guaporensis* 96,52 %	KY268181	-	-	
LRA 377	P3	*Makunaima guaporensis* 96,52 %	KY268181	-	-	

Catalog Number	Point	GenBank (% similarity)	Accession code	BOLD (% similarity)	Accession code	Morphological ID
LRV 367	P3	*Astyanax bimaculatus* 100 %	KY267142	*Astyanax bimaculatus* 99,84 %	BAST1482-14	*Astyanax* aff. *argyrimarginatus*
LRV 368	P3	*Astyanax bimaculatus* 98,21 %	KY268038	*Astyanax bimaculatus* 98,19 %	BAST1274-13	*Astyanax bimaculatus*
LRV 369	P3	*Astyanax bimaculatus* 98,19 %	KY268038	*Astyanax bimaculatus* 98,18 %	BAST1274-13	
PLAG 337	P3	*Apareiodon affinis* 99,12 %	AP011998	*Apareiodon affinis* 98,90 %	GBMTG1926-16	*Apareiodon* sp.
PLAG 338	P3	*Apareiodon affinis* 99,65%	AP011998	*Apareiodon affinis* 99,30%	GBMTG1926-16	
PLAG 339	P3	*Apareiodon affinis* 98,21 %	AP011998	*Apareiodon affinis* 98,20 %	GBMTG1926-16	
PLAG 340	P3	*Apareiodon affinis* 99,35 %	AP011998	*Apareiodon affinis* 99,17 %	GBMTG1926-16	
PMTA 371	P3	*Astyanax bimaculatus* 99,65%<br>*Psellogrammus kennedyi* 98,26%	KY268241<br>JN989172	*Ctenobrycon hauxwellianus* 99,82 %<br>*Astyanax bimaculatus* 99,48%<br>*Ctenobrycon* sp. 99,44%<br>*Psellogrammus kennedyi* 98,40%<br>*Astyanax* sp. 98,22%<br>*Ctenobrycon alleni* 98,04%<br>*Jupiaba acanthogaster* 98,04%	PRIVATE<br>BAST1275-13<br>PRIVATE<br>PRIVATE<br>EARLY RELEASE<br>PRIVATE<br>PRIVATE	*Ctenobrycon spilurus*

Catalog Number	Point	GenBank (% similarity)	Accession code	BOLD (% similarity)	Accession code	Morphological ID
PMTA 373	P3	*Astyanax bimaculatus* 99,51 %<br>*Psellogrammus kennedyi* 98,20%	KY268241<br>JN989172	*Ctenobrycon hauxwellianus* 99,66 %<br>*Astyanax bimaculatus* 99,34%<br>*Ctenobrycon* sp. 99,30%<br>*Psellogrammus kennedyi* 98,32%<br>*Astyanax* sp. 98,16%	PRIVATE<br>BAST1275-13<br>PRIVATE<br>PRIVATE<br>EARLY RELEASE	
PMTA II416	P3	*Astyanax bimaculatus* 99,67 %<br>*Psellogrammus kennedyi* 98,20%	KY268241<br>JN989172	*Ctenobrycon hauxwellianus* 99,83 %<br>*Astyanax bimaculatus* 99,67 %<br>*Ctenobrycon* sp. 99,37%<br>*Psellogrammus kennedyi* 98,49%<br>*Astyanax* sp. 98,32%<br>*Ctenobrycon alleni* 98,16%<br>*Jupiaba acanthogaster* 98,16%	PRIVATE<br>BAST1275-13<br>PRIVATE<br>PRIVATE<br>EARLY RELEASE<br>PRIVATE<br>PRIVATE	
AAA403	P3	*Caquetaia spectabilis* 98,37%	OR732852	*Caquetaia spectabilis* 99,25%	ANGBF7530-12	*Caquetaia spectabilis*
AAA404	P3	*Caquetaia spectabilis* 98,91%	OR732852	*Caquetaia spectabilis* 99,81%	ANGBF7530-12	
ARV408	P3	*Pseudanos trimaculatus* 98,60%	KF569003	*Pseudanos trimaculatus* 98,77%	PRIVATE	*Pseudanos gracilis*
PBP 409	P3	*Chilodus punctatus* 98,74%	KF562431	*Chilodus punctatus* 97,91%	AMCC095-06	*Chilodus punctatus*
RFO326	P3	*Aphyocharax anisitsi* 87,46%	JN988679	-	-	*Aphyocharax* sp.
RFO327	P3	*Aphyocharax anisitsi* 87,46%	JN988679	-	-	
RFO328	P3	*Aphyocharax anisitsi* 87,46%	JN988679	-	-	
RFO329	P3	*Aphyocharax anisitsi* 87,46%	JN988679	-	-	

**Catalog Number**	**Point**	**GenBank (% similarity)**	**Accession code**	**BOLD (% similarity)**	**Accession code**	**Morphological ID**
TAZ 344	P3	*Serrapinnus notomelas* 96,73%	HM126637	*Serrapinnus* aff. *notomelas* 99,66%	PRIVATE	*Serrapinnus* cf. *notomelas*
TAZ 345	P3	*Serrapinnus notomelas* 96,73%	HM126637	*Serrapinnus* aff. *notomelas* 99,66 %	PRIVATE	
TAZ 346	P3	*Serrapinnus* sp. 96,72%	HM126637	*Serrapinnus* aff. *notomelas* 99,66%	PRIVATE	
TOF 389	P3	*Moenkhausia oligolepis* 91,56%	MN172486	-	-	*Bario oligolepis*
